# Supplementary material for: Exploratory Pilot Study of Circulating Biomarkers in Metastatic Renal Cell Carcinoma
Source: Cancers (Basel). 2020 Sep 14;12(9):2620. doi: 10.3390/cancers12092620 (PMC7563741; doi:10.3390/cancers12092620)
Supplement: Supplementary file 1 [file cancers-12-02620-s001.pdf]

Supplementary Materials: Exploratory Pilot Study of Circulating Biomarkers in Metastatic Renal Cell Carcinoma

Ilaria Grazia Zizzari, Chiara Napoletano, Alessandra Di Filippo, Andrea Botticelli, Alain Gelibter, Fabio Calabrò, Ernesto Rossi, Giovanni Schinzari, Federica Urbano, Giulia Pomati, Simone Scagnoli, Aurelia Rughetti, Salvatore Caponnetto, Paolo Marchetti and Marianna Nuti

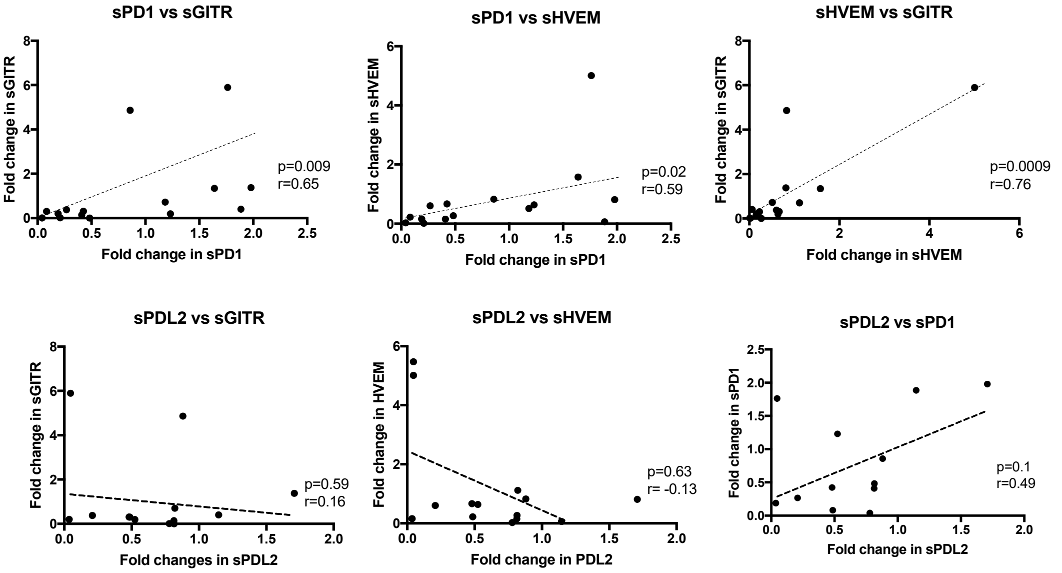

Figure S1. Correlations between the fold changes (T>0/T0) of sIC molecules modulated by TKI treatment in mRCC.

Table S1. MoAbs used in this study.

Antigen	MoAb Clone	Catalog Number	Supplier
CD3 (APC-H7)	UCHT1	300456	Biolegend
CD8 (PerCp-Cy5.5)	RPA-T8	560662	BD Biosciences
CD45RA (BB515)	HI100	564552	BD Biosciences
CD4 (APC-H7)	RPA-T4	560158	BD Biosciences
CD25 (PE)	M-A251	356134	Biolegend
FoxP3 (Alexa647)	259D/C7	560045	BD Biosciences
CD137 (PeCy7)	4B4-1	309818	Biolegend
PD-1 (PE)	MIH4	557946	BD Biosciences
CTLA4 (APC)	BNI3	555855	BD Biosciences
Tim3 (BB515)	7D3	565568	BD Biosciences
IgG (PerCp-Cy5.5)	MOPC-21	550795	BD Biosciences
IgG (PeCy7)	MOPC-21	557872	BD Biosciences
IgG (BB515)	X40	564416	BD Biosciences
IgG (Alexa647)	MOPC-21	557732	BD Biosciences
IgG (APC)	MOPC-21	550854	BD Biosciences
IgG (APC-H7)	27-35	560183	BD Biosciences
IgG1 (PE)	MOPC-21	400112	Biolegend

Table S2. Others sIC molecules and cytokines/chemokines analyzed in the study.

	**TKI treatment**		
Soluble molecules	**T0** (pg/mL)	**>T0** (pg/mL)	***p*** (T0 vs >T0)
CD80	1405±1341	503±394	*0.08*
CTLA4	585±410	457,65±324	*0.2*
PD-L1	107±92	75±73	*0.4*
Tim3	5352±3265	3688±2441	*0.1*
BTLA4	366±516	875±938	*0.2*
CD27	12974±11460	10012±11068	*0.2*
CD28	2856±2390	1849±1288	*0.2*
IDO	99±65	74±50	*0.1*
LAG3	472±290	384±175	*0.7*
IL-1alpha	7±10	6±5	*0.8*
IL-1beta	4±5	17±27	*0.1*
IL-4	88±130	200±298	*0.9*
IL-8	6±10	12±21	*0.1*
Il-12p70	179±145	221±193	*0.5*
IL-13	15±16	16±18	*0.6*
IL-17	59±79	21±22	*0.1*
IFN-alpha	6±8	9±15	*0.4*
TNF-alpha	172±137	166±144	*0.5*
sICAM-1	140671±98168	184281±160311	*0.07*
MIP-1alpha	30±25	26±21	0.9
MIP-1beta	57±39	71±59	*0.7*
E-Selectin	45626±21566	37859±19484	*0.1*
P-Selectin	159935±120805	138666±117891	*0.9*

© 2020 by the authors. Licensee MDPI, Basel, Switzerland. This article is an open access article distributed under the terms and conditions of the Creative Commons Attribution (CC BY) license (http://creativecommons.org/licenses/by/4.0/).
